# Caregiver‐reported increased food motivation and adiposity in dogs receiving antiseizure drugs

**DOI:** 10.1002/vetr.4907

**Published:** 2024-12-10

**Authors:** Anna Morros‐Nuevo, Rowena M. A. Packer, Nicole Regan, Eleanor Raffan

**Affiliations:** ^1^ Department of Physiology Development and Neuroscience University of Cambridge Cambridge UK; ^2^ Department of Clinical Science and Services Royal Veterinary College Hatfield UK

**Keywords:** dogs, epilepsy, obesity, owner attitudes to pets, veterinary profession

## Abstract

**Background:**

Idiopathic epilepsy is the most common chronic neurological disease in dogs and requires lifelong administration of antiseizure drugs (ASD). A decreased level of energy and increased food intake and weight gain have been described as long‐lasting side effects.

**Methods:**

We assessed food motivation (FM), using the previously validated dog obesity risk assessment questionnaire, in dogs diagnosed with idiopathic epilepsy (*n* = 222) and healthy dogs (*n* = 7086) to determine if epilepsy and ASD were associated with increased FM and adiposity and decreased activity. We also assessed how caregivers managed weight gain in this population of dogs in the study.

**Results:**

Dogs with idiopathic epilepsy receiving ASD had significantly higher FM than healthy dogs. Their carers also reported significantly greater interventional effort and food restriction compared with healthy dogs, yet they had significantly higher adiposity. Minimal modelling showed that within the epileptic group, ASD had the highest impact on FM, with an effect size of 32%.

**Limitations:**

Carer‐reported data were used, which could have introduced bias. Furthermore, the sample size did not allow us to distinguish the effect of individual ASD.

**Conclusions:**

ASD increases FM in dogs, resulting in greater adiposity.

## INTRODUCTION

Idiopathic epilepsy (IE) is the most common chronic neurological disease in dogs, with an estimated prevalence of 0.6%.^1^ Historically, the primary aim of treating dogs with epilepsy has been reducing the frequency and severity of seizures, which is most commonly achieved with the use of antiseizure drugs (ASD).^2^ Despite this goal, ASD therapy rarely leads to seizure freedom,^3^ and many dogs receiving long‐term ASD treatment experience adverse effects during the interictal period. In recent studies, over 80% of caregivers of dogs administered ASD reported ataxia, lethargy and increased thirst and appetite.^4^ Such effects are more commonly reported in patients treated with a combination of more than one ASD (polytherapy)^4^, ^5^, ^6^ and are a key concern for owners of epileptic dogs.^7^ Furthermore, given that the probability of seizure control diminishes progressively with successive ASD treatments,^8^ postictal signs (e.g., restlessness and temporary blindness), which have a negative impact on quality of life (QoL), might not be deterred by the use of ASD.^9^

In people with epilepsy, ASD adverse effects are negatively associated with self‐reported QoL, having a greater impact than seizure frequency.^10^ Similarly, in dogs with epilepsy, data suggest that caregiver‐reported QoL scores are significantly lower in dogs experiencing ASD adverse effects than those that are not.^4^, ^11^ To reliably address ASD adverse effects in research (e.g., drug development and clinical trials) and in practice (e.g., finding a balance between seizure control and adverse effect severity), methods for objective quantification of adverse effects are required.

Increased interest in food is a frequently cited ASD adverse effect.^12^ This has commonly been referred to in the literature as ‘polyphagia’ or ‘hyperphagia’, terms that strictly imply increased food intake but are commonly used in the veterinary literature to describe excessive food drive or appetite,^13^, ^14^ largely because there has not been a well‐defined measure of the drive to eat in dogs.

An increased desire to eat (where ad libitum food is not available, as in most homes where pet dogs are kept) has been proposed as a source of primary, negative emotions,^14^ which may impact QoL if it becomes chronic. Excessive drive to eat may lead to undesirable behaviours such as begging and scavenging, which can have a negative impact on the dog‒caregiver bond.^15^, ^16^ In addition, increased food drive or food motivation (FM) may also lead to further medical issues related to overeating and obesity, especially in combination with reduced physical activity, which is another common ASD side effect.^15^, ^16^, ^17^, ^18^ In turn, obesity is associated with reduced lifespan and poor QoL^19^, ^20^ and predisposes individuals to health conditions including orthopaedic and respiratory diseases, urinary incontinence and dyslipidaemia.^21^, ^22^, ^23^, ^24^, ^25^, ^26^, ^27^

Despite the potential consequences of ASD‐related increased FM for dogs and their caregivers, to the authors' knowledge, there are no published attempts to quantify this common adverse effect or understand caregiver management of weight and FM in dogs with epilepsy. This likely reflects having limited tools to measure such effects and differences in their perceived importance between stakeholder groups. For example, in a recent study, caregivers and veterinarians working in general practice ranked adverse effects of ASD and why they occur as a higher priority research area for canine epilepsy than specialist veterinary neurologists,^28^ a finding that has also been observed in human medicine.^29^

We previously developed a validated measure of canine FM and owner management practices: the dog obesity risk assessment (DORA) questionnaire. The questionnaire asks dog caregivers to respond to a series of statements about their dogs’ behaviour around food and their management of diet and exercise using a Likert scale. It has been shown to be repeatable and suitable for administration at scale, enabling systematic use in large clinical populations. A high ‘food motivation score’ (FMS, scale 0‒1) suggests that dogs persistently seek food in the home environment through both proximity and ‘begging’‐type behaviours.^30^ The use of the questionnaire has contributed to evidence that highly food‐motivated dogs are at risk of developing obesity and that FM varies between breeds and with age, sex and neuter status.^30^, ^31^

We hypothesised that (1) the previously reported ‘hyperphagia’ or ‘polyphagia’ in dogs receiving ASD would be quantifiable as an increase in FMS relative to control dogs; (2) that ASD polytherapy would be related to a greater increase in FM than monotherapy; and (3) that owner management efforts to control weight will increase with increasing dog FM.

## METHODS

### Data collection and curation

This project was approved by the Social Science Research Ethical Review Committee at the Royal Veterinary College, London, UK. Caregivers of epileptic dogs aged between 1 and 20 years were recruited online via social media platforms and forums between July and August 2018. Caregivers of healthy (control) dogs were recruited through the same method between May 2015 and October 2021.

#### Inclusion and exclusion criteria

For both groups, the following exclusion criteria were applied:

1. Dogs that suffered medical conditions other than IE or were taking medications other than ASD that could affect weight, FM or activity levels were excluded. These medications included, but were not limited to, corticosteroids and medications used to treat thyroid disease, diabetes and hyperadrenocorticism (Supporting Information, Section 1).
2. Body condition score (BCS, 1–9) is a measure of canine adiposity based on haptic and visual cues,^32^, ^33^ where 5 represents ideal bodyweight and every point above that corresponds to an ∼8% increase in adiposity.^33^, ^34^ Dogs with a BCS of 3 or lower were excluded, as this value is considered underweight and may be indicative of underlying health conditions (Supporting Information, Section 3).

To be included in the epileptic group, dogs had to meet Tier I confidence level for the diagnosis of IE (hereafter referred to as the ‘epileptic group’); that is a history of two or more unprovoked epileptic seizures occurring at least 24 hours apart (in some but not all cases including review of video footage of the seizures by a veterinarian, which is consistent with the Tier 1 criteria), an age between 6 months and 6 years when the first seizure occurred, and normal neurological examination, blood tests and urinalysis that show no discernible cause for the seizures.^2^

#### Matched control selection

The control group was selected at random except that we enriched for breeds which were most commonly represented in the case group, because breed has previously been associated with food motivation.^30^, ^35^, ^36^ Despite this, some breeds were still more common in the case compared to the control group. We note that the distribution of sex and neuter status was similar between control and case groups, which is important because those factors are also associated with food motivation.^31^, ^37^, ^38^, ^39^, ^40^, ^41^, ^42^, ^43^, ^44^, ^45^, ^46^

#### Survey design

The participants were invited to complete an online survey published through commercially available software,^47^ which was structured in four sections (Supporting Information, Section 2):

- Signalment and clinical history
- DORA questionnaire
- Identification of their dog's BCS from a visual scale
- Perceived ASD impact on behaviour and FM (epileptic group only)

### Statistical analysis

Statistical analysis was performed with R Studio (version 4.3.1).^48^ Univariate statistical analysis with Holm correction was used to assess differences in age, BCS and DORA scores between groups (epileptic vs. control) and, within the epileptic group, between drug‐naïve dogs and those receiving monotherapy or polytherapy. Pearson's correlations were used to analyse how side effects, BCS and FMS correlated with the number of ASD taken. To assess the combined effect of ASD and factors predicted to alter eating behaviour within the epileptic group, minimal modelling was performed using Akaike's information criterion (Supporting Information, Section 4).

## RESULTS

### Population features

Two hundred and twenty‐two dogs of various breeds were included in the epileptic group (Table S2), of which 86 were female (11.6% entire and 88.4% neutered) and 136 were male (25.7% entire and 74.3% neutered). From an initial database of over 19,000 responses, the final number of subjects remaining in the control group was 7086 after applying inclusion and matching criteria, of which 3036 were female (11.7% entire and 88.3% neutered) and 4050 were male (30.9% entire and 69.1% neutered). The mean age (± standard deviation [SD]) did not differ significantly between epileptic and control groups (5.50 ± 3.58 years and 5.64 ± 4.78 years, respectively). Crossbred dogs were the most common type in both the epileptic (24.77%) and control groups (25.15%). Border Collies and ‘other purebreds’ were overrepresented in the epileptic group (16.67% and 10.81%, respectively) compared with the control group (6.46% and 7.30%, respectively).

Within the epileptic group, eight (3.6%) dogs were drug naïve, 80 (36%) received monotherapy and 134 (60.4%) received polytherapy. The most common ASD used within the epileptic group was phenobarbital (*n* = 179, 80.6%), followed by potassium bromide (*n* = 90, 40.5%), levetiracetam (*n* = 83, 37.4%), imepitoin (*n* = 27, 12.2%), zonisamide (*n* = 21, 9.5%), gabapentin (*n* = 8, 3.6%), clorazepate (*n* = 4, 1.8%) and topiramate (*n* = 1, 0.5%). The mean age (± SD) at the onset of seizures was 2.68 ± 1.51 years. The mean duration of treatment (± SD) varied by drug, with levetiracetam having the shortest duration (15.6 ± 18.1 months) and phenobarbital the longest (86.1 ± 76.1 months).

### BCS, FMS and owner management factors

**FIGURE 1:**
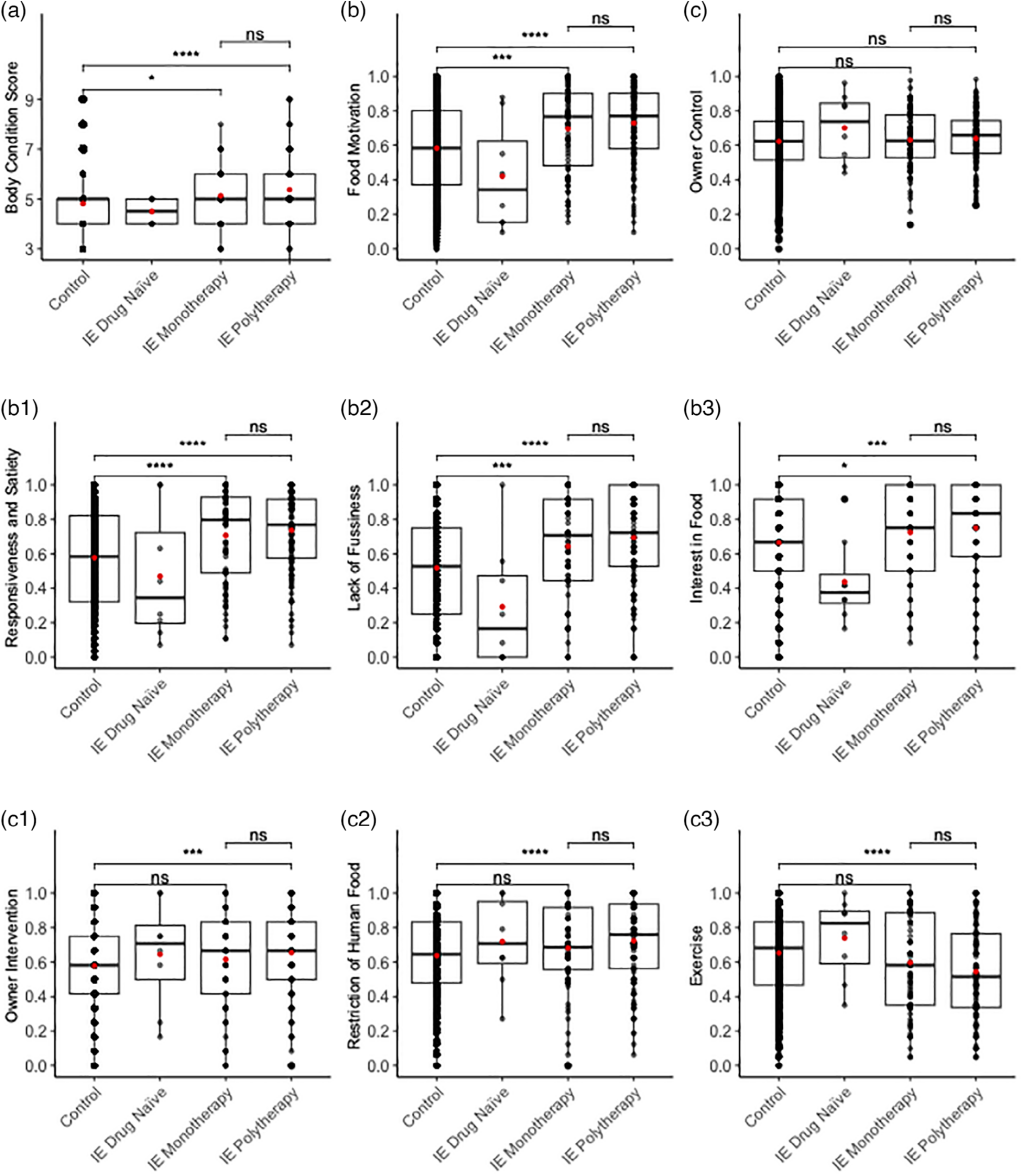
Box and whiskers plots showing body condition scores (a) and dog obesity risk assessment (DORA) scores for food motivation (b) and its subcomponents (b1‒b3) and owner control (c) and its subcomponents (c1‒c3) in healthy control dogs, drug‐naive dogs with idiopathic epilepsy (IE), dogs with IE receiving antiseizure drug (ASD) monotherapy and dogs with IE receiving ASD polytherapy. The box represents upper and lower quartile values with the median shown in the middle and the mean represented with a red dot. The vertical line represents minimum and maximum data value and individual scores and are shown as black dots. The level of significance (*t*‐test) is shown as ‘ns’ (*p* > 0.05), ^*^ *p* < 0.05, ^**^ *p* < 0.01 or ^***^ *p* <0.001

Compared to the control group, epileptic dogs receiving ASD had significantly higher BCS (*p* < 0.001), FMS (*p* < 0.001) and FMS subcomponents: responsiveness and satiety (*p* < 0.001), lack of fussiness (*p* < 0.001) and interest in food (*p* < 0.001). Epileptic dogs also had significantly higher owner intervention scores (*p* < 0.002) and restriction of human food scores (*p* < 0.001) and significantly lower exercise scores (*p* < 0.001). The overall owner control score, which combines the intervention score, restriction of human food score and exercise score, did not differ significantly between groups (Figure 1; Supporting Information, Section 6, Tables S3 and S4).

### ASD side effects and dietary management

**FIGURE 2:**
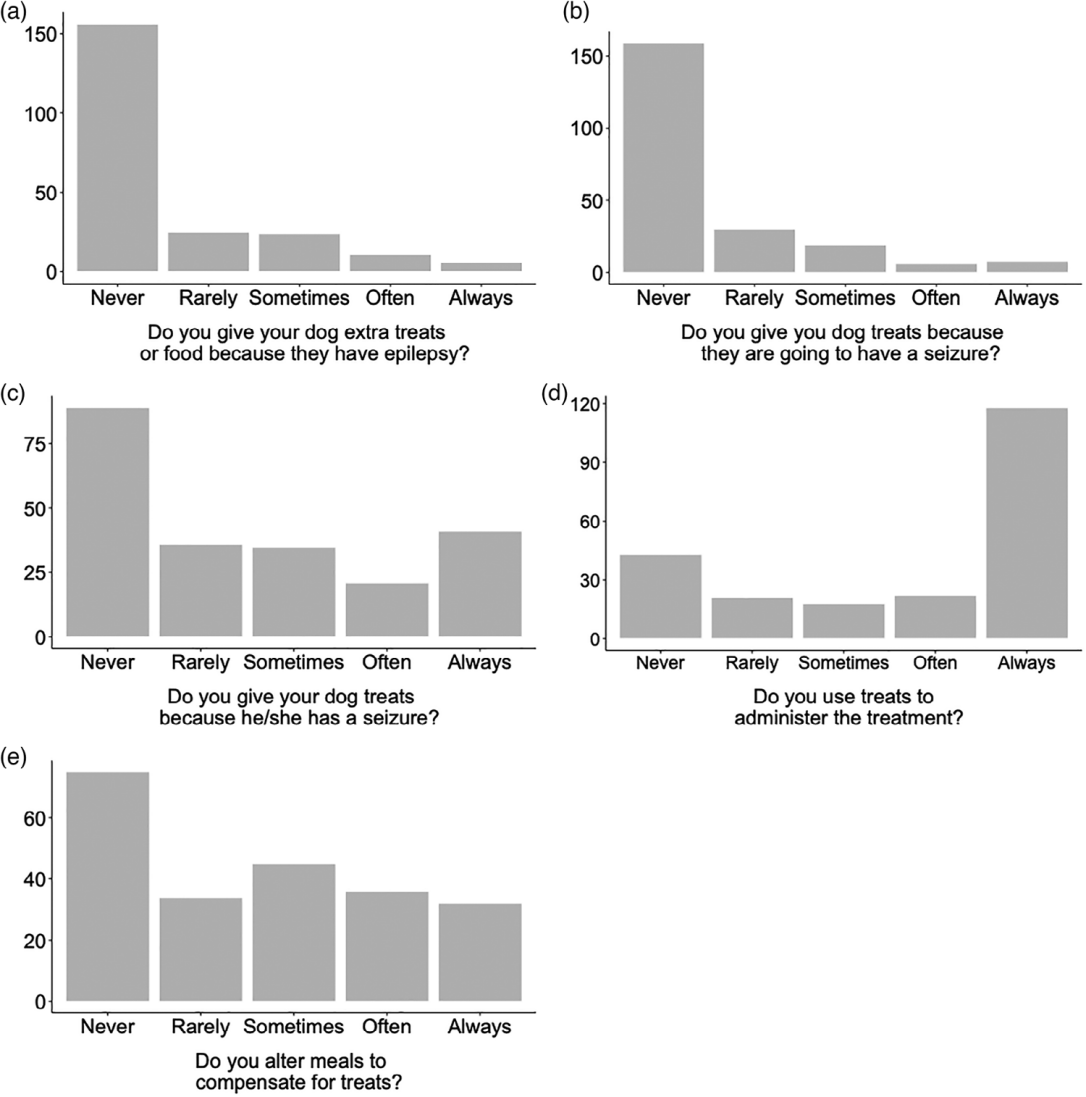
Bar plots showing the number of participants who reported ‘never’, ‘rarely’, ‘sometimes’ ‘often’ or ‘always’ (a) giving extra treats to their dogs because they have epilepsy, (b) giving treats because they are going to have a seizure, (c) giving treats because they had a seizure, (d) using treats to administer ASD and (e) altering meals to compensate for treats

Most caregivers reported never using extra treats because their dog had epilepsy (70.0%) or giving extra treats before (71.6%) or after (40%) their dog had a seizure. However, many always used treats to administer their dog's ASD medication (53.0%) and did not compensate for it by reducing the dog's main food ratio (33.8%) (Figure 2; Table S5).

**FIGURE 3:**
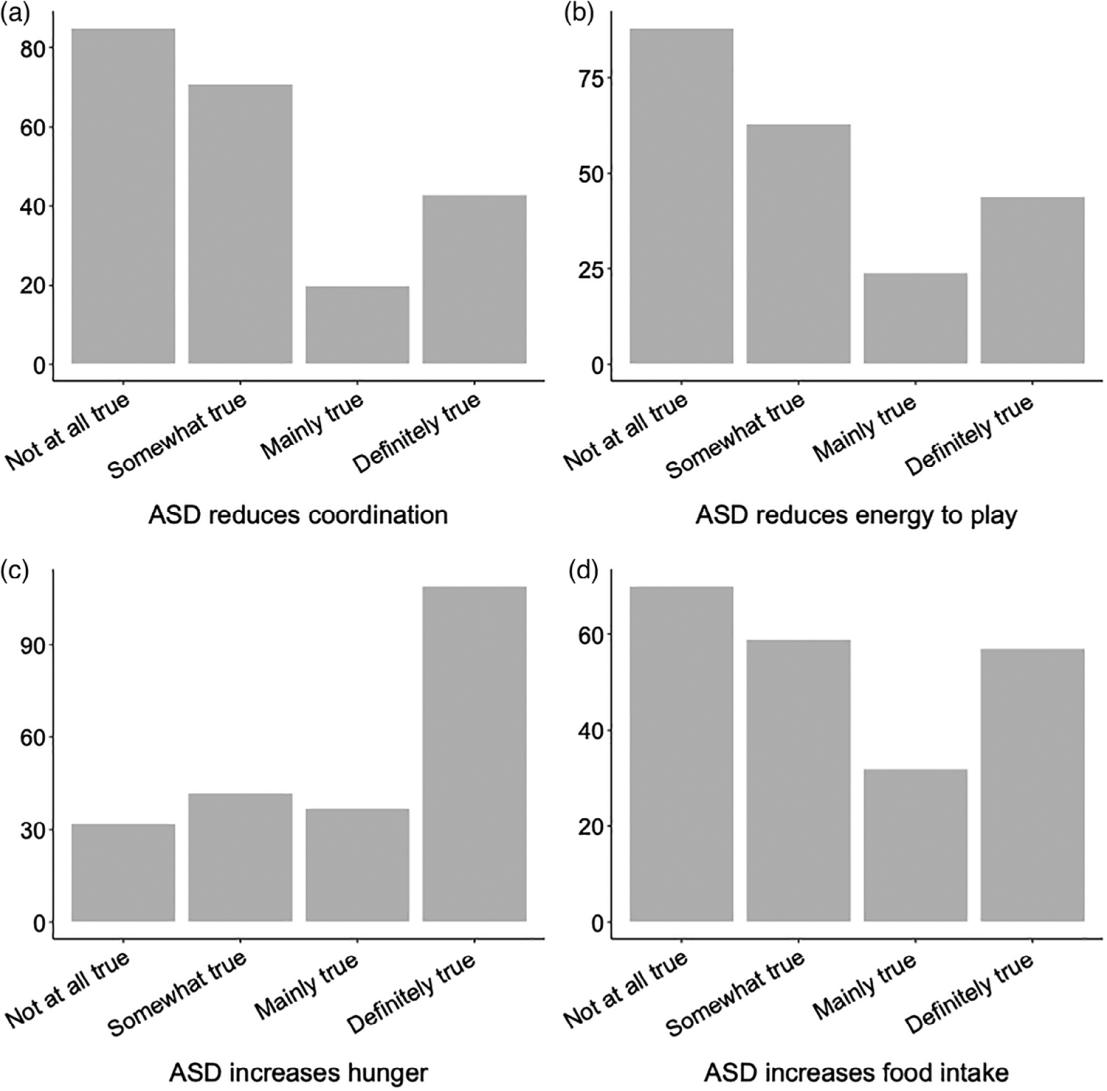
Bar plots showing the number of participants who considered it ‘not at all true’, ‘somewhat true’, ‘mainly true’ or ‘definitely true’ that ASD (a) resulted in a decrease in their dog's coordination, (b) decreased level of energy to play, (c) increased hunger or (d) increased food intake

Phenobarbital was most commonly reported as being associated with side effects (*n* = 92/179, 51%), followed by imepitoin (*n* = 8/27, 29%), potassium bromide (*n* = 19/90, 21%), zonisamide (*n* = 4/21, 19%) and levetiracetam (8/83, 9%). Most respondents considered increased hunger as a side effect of ASD but not increased food intake (Figure 3; Table S6).

### ASD, FMS and obesity

There were only eight drug‐naïve epileptic dogs (four Border Collies, one Boston Terrier, two crossbreeds and one West Highland White Terrier), and they showed no significant differences in BCS and FMS when compared with control dogs. However, the control group had significantly higher lack of fussiness (*p* = 0.036) and interest in food (*p* = 0.012) scores. Negative results were to be anticipated due to the analysis being underpowered, with a post hoc power calculation showing that we would require 20 times more drug‐naïve epileptic dogs to detect small differences between the two populations.^49^, ^50^ The unequal sample sizes might reduce power because smaller samples provide less precise estimates of group means, and the *t*‐statistic becomes less sensitive to true mean differences due to the weight of the smaller sample size.^51^, ^52^ However, we compared drug‐naïve epileptic dogs against all epileptic dogs receiving ASD and found that dogs receiving ASD had significantly higher FMS (*p* = 0.002) and its subcomponents: responsiveness and satiety (*p* = 0.022), lack of fussiness (*p* = 0.001) and interest in food (*p* = 0.002). There were no significant differences in BCS and age (Figure 1; Tables S3 and S4).

We then compared epileptic dogs receiving monotherapy with those receiving polytherapy and found no significant differences (Figure 1; Tables S3 and S4). Since this binary classification of monotherapy versus polytherapy is crude, we also considered whether the number of drugs might be significant. The number of drugs used varied considerably (0 ASD: *n* = 8; 1 ASD: *n* = 80; 2 ASD: *n* = 82; 3 ASD: *n* = 40; 4 ASD: *n* = 11; 5 ASD: *n* = 1) and was significantly positively correlated with carers' reports of decreased energy (*r* = 0.30, *p* < 0.001), decreased coordination (*r* = 0.43, *p* < 0.001) and increased owner perception of dogs’ hunger (*r* = 0.18, *p* = 0.007). It was not significantly correlated with perceived increase in food intake, FMS, responsiveness and satiety score and interest in food score, but it was significantly but weakly positively correlated with BCS (*r* = 0.14, *p* = 0.032) and lack of fussiness score (*r* = 0.15, *p* = 0.026).

### Sex, neuter status and ASD explain the majority of variability in FMS

Within the epileptic group, the minimal model for FMS (initially containing sex, age and neuter status, allowing two‐way interactions) retained sex and neuter status as significant variables, which explained 1.8% of the variability. Adding ASD (receiving ASD: yes and no) to the model increased variability explained to 8.9%, and ASD remained significant (*β* = 0.32, *p* < 0.001), alongside sex (*β* = ‒1.17, *p* = 0.042) and the interaction between sex and neuter status (*β* = 0.23, *p* = 0.012) (Table S1).

## DISCUSSION

This survey of the effect of epilepsy and ASD on FM in dogs identified that epileptic dogs receiving ASD tend to be more food motivated. The data suggest that ASD are responsible for the changes shown, not epilepsy per se, although the study was underpowered to prove that. We further show that caregivers of dogs receiving ASD tend to restrict their dogs’ access to food more assiduously than caregivers of non‐epileptic dogs, but despite this, epileptic dogs receiving ASD tend to have greater BCS.

Our data are consistent with previous reports that treatment with ASD is associated with increased food drive, hunger or similar terms reported across different studies.^53^ A previous review found that 29‒44% of studies reported polyphagia in dogs treated with one of a number of commonly used ASD.^53^ Another systematic review of the safety of potassium bromide found that 15% of publications provided primary evidence regarding polyphagia as an adverse effect in dogs.^54^ However, such studies provide no detail on the percentage of all reviewed dogs affected and tend to use unvalidated measures of food intake, food drive and hunger. Our data capitalised on a previously validated measure of canine eating behaviour^30^ and benefitted from a large, breed‐matched control population; therefore, our findings robustly demonstrate that ASD are associated with greater FM in the home environment.

The authors acknowledge that the inclusion of IE dogs over a wide age range (1‒20 years) means that some dogs may have undiagnosed comorbidities that could affect the data. Similarly, despite all IE dogs having Tier 1 diagnoses according to the International Veterinary Epilepsy Task Force consensus report on epilepsy definition, it is possible that some might have pathologies which can occasionally meet these criteria, and information about the onset of the first seizure at the time of diagnosis is lacking. However, despite these possible confounders, the results remain clear. A common limitation in ASD studies is the difficulty of distinguishing which is the causative drug when it is used as part of polytherapy. Chang et al.^7^ found that polyphagia was 30% higher in dogs receiving phenobarbital monotherapy than potassium bromide monotherapy. However, in dogs receiving a combination of the two drugs, the results are less consistent, suggesting that it is a combined effect of both drugs that results in increased ‘polyphagia’. Similarly, in our population, most participants considered that phenobarbital affected appetite the most, followed by potassium bromide. However, those were also the most common ASD, and the study was insufficiently powered to statistically compare the effect of individual ASD on FM and adiposity. When we compared epileptic dogs receiving ASD monotherapy or polytherapy as a binary characteristic, our data showed no significant differences in BCS, FMS and its subcomponents, but the number of ASD was positively correlated with a perceived decrease in energy and coordination, increased hunger but not food intake, and decreased lack of fussiness score, suggesting that polytherapy might be associated with greater side effects. This was a secondary analysis, and the study was not designed to test the effect of monotherapy versus polytherapy on FM and obesity, so the absence of other significant differences does not rule out that they might exist.

Likewise, it seems unlikely that dogs receiving ASD who are reported to have increased hunger do not have a parallel increase in food intake, as owners have been shown to perform poorly at limiting food availability.^55^ This trend is further impacted by the fact that a majority of dog carers rely on food to help administer their dog's ASD medication. Epileptic dogs treated with ASD commonly receive oral medication every 8‒12 hours, which has a potentially large accumulative impact on calorie input. Furthermore, a recent study^56^ found that a large proportion (45.8%) of carers of dogs with epilepsy give their dog dietary supplements in an attempt to manage their epilepsy, with these supplements commonly belonging to the fatty acid group, which is highly calorific. Cumulatively, these attempts to control seizure frequency and/or severity could result in a large daily intake of calories in addition to these dogs' base diets.

Although polyphagia is associated with commonly administered ASD,^4^ it is sometimes considered transient, with reports suggesting it to be at greatest intensity in the first 2‒4 weeks of treatment and subsiding after this period once serum levels reach a steady state^57^, ^58^, ^59^ (although that is not always the case^60^). Our cross‐sectional dataset did not allow us to identify longitudinal changes over time, but a previous study by Boothe et al.^57^ compared the proportion of dogs exhibiting polyphagia 1 and 6 months after starting treatment and identified a significant decrease in proportion over that time, from 30% to 0% for dogs receiving phenobarbital and 39% to 4.3% for dogs receiving potassium bromide. This is in accordance with another study^5^ where polyphagia was found to be the most common and severe side effect reported, and on average, it reduced in severity after the first 2 weeks. That study also revealed that the median score of polyphagia was greater in polytherapy than in monotherapy cases, supporting our findings that increased numbers of ASD were significantly positively correlated with increased perceived hunger, lack of fussiness (related to food) and BCS. Overall, our results empirically corroborate existing data that ASD therapy increases FM in dogs, even after a protracted treatment period.

### Epilepsy and obesity

Dogs with epilepsy are potentially predisposed to become obese not only due to the combined effects of polyphagia and reduced activity (sedation) from ASD but possibly because of the physical and psychological stress caused by the condition itself.^9^, ^61^ In a survey of caregivers, it was found that dogs with chronic poor health showed more signs of stress‐induced emotional eating.^55^ However, it is possible that these participants were required to pay more attention to their dog's diet and feeding habits, resulting in inaccurately reported emotional eating. Rowland and Antelman^62^ demonstrated that, in rats, polyphagia can be induced by chronic stress (mild tail pinches several times a day) and, through analysis of hair cortisol levels, dogs with epilepsy have been shown to experience chronic stress compared to non‐epileptic dogs. Epileptic dogs receiving ASD might also suffer from chronic hunger, contributing to chronic stress levels. Indeed, a study by Sandilands et al.^63^ found that blood indices of stress were higher in broiler chickens experiencing chronic hunger due to feed restriction, and there is evidence of increased glucose utilisation within the brain during and immediately after a seizure in people,^64^ which may, in turn, contribute to increased hunger. Only dogs with a BCS of more than 4 were included in our study, which aimed to exclude dogs in which comorbidities or persistent seizures might confound the effect of ASD on eating behaviour because their low body condition might be driving hyperphagia or be a reflection of poor appetite related to systemic conditions. We believe that our data are representative of the most common form of IE and may provide a higher BCS estimate population wide.

In people, obesity and epilepsy are comorbid, with several mechanisms for this association proposed, including (1) ASD stimulating appetite; (2) reduced physical activity of epileptic patients; (3) seizures disrupting hormone systems related to energy intake, metabolism and expenditure^65^; and (4) obesity priming the brain for seizures.^65^ In accordance with previous studies,^4^ our data support that dogs receiving ASD tend to have higher BCS, which might be in part mediated by increased FM and decreased exercise levels.^66^ However, due to the small sample size, the effect of epilepsy and ASD cannot be distinguished in our sample population. Lethargy is a common side effect of ASD treatment.^12^ It is therefore also possible that as well as being a method of coping with stress, dogs with epilepsy are eating more due to boredom/frustration caused by lethargy. Furthermore, exercise has also been suggested to have an effect on reducing stress. Unsurprisingly, in our epileptic population, Border Collies were overrepresented,^4^ but interestingly, this breed has a low average FMS and obesity probability and tends to be more active^67^; however, this group had significantly higher FMS and BCS and significantly lower exercise levels than healthy dogs. Given that owner‐reported BCS was used in this study, which is known to be biased towards a more ideal value,^68^, ^69^, ^70^, ^71^, ^72^ it is likely that BCS differences are underestimated.

Previous studies have reported risk factors for obesity such as sex, neuter status, age and breed.^31^, ^37^, ^38^, ^39^, ^40^, ^41^, ^42^, ^43^, ^44^, ^45^, ^46^, ^73^ Our results showed that sex, age and neuter status explained less than 2% of the variability in FM within the epileptic group. However, when ASD (receiving or not receiving ASD) was added to the model, the variability explained increased to 8.9%, and the use of ASD had the largest significant positive effect, with the potential to increase FMS by 32%. Unfortunately, this model only contained eight drug‐naïve epileptic dogs; therefore, these results must be interpreted carefully.

### Epilepsy and owner management of weight and appetite

Owners tend to underestimate their pets' weight status^68^, ^69^, ^70^, ^71^, ^72^ and overestimate exercise levels.^74^, ^75^ White et al.^72^ reported that only half of the dog owners they surveyed agreed with their vet on the degree to which their pet was overweight; while some recognised that their dog had a weight problem, they preferred to classify it as overweight rather than obese. However, differences between epileptic and healthy dogs are being identified by our participants. The epileptic dogs in our study were perceived as exercising significantly less and having significantly greater FM and significantly higher adiposity. Our data also show that owners of epileptic dogs apply more self‐reported food restrictions and interventional efforts than owners of healthy dogs, but the overall management remains the same. This finding suggests that owners of epileptic dogs receiving ASD recognise that their dogs are more food motivated and less active and try to compensate for it by managing their diet. Nonetheless, the differences in BCS show that they fail in their efforts to keep their dog's weight down, in accordance with previous findings in the general canine population.

## CONCLUSION

In conclusion, dogs taking ASD are more food motivated and have greater adiposity than healthy dogs. Dogs with epilepsy are also reported to exercise less, and although caregivers compensate for this by restricting food more, their overall efforts to control weight appear to be ineffective. Sources of additional calories that may be thwarting owners' attempts to control their dog's weight were identified, particularly using treats to administer dogs’ ASD twice or more per day. Given the potential negative impacts of obesity, owners of dogs with epilepsy should be counselled on the impact of ASD on FM when first offered as treatment and should be offered support (e.g., weight clinics) to monitor their dog's BCS while receiving ASD.

## AUTHOR CONTRIBUTIONS

*Conceptualisation and design of the work*: Rowena M.A. Packer, Anna Morros‐Nuevo and Eleanor Raffan. *Data acquisition*: Nicole Regan, Rowena M.A. Packer and Eleanor Raffan. *Methodology*: Anna Morros‐Nuevo, Rowena M.A. Packer and Eleanor Raffan. *Data analysis*: Anna Morros‐Nuevo and Nicole Regan. *Interpretation of data*: Anna Morros‐Nuevo, Rowena M.A. Packer, Nicole Regan and Eleanor Raffan. *Visualisation*: Anna Morros‐Nuevo and Nicole Regan. *Drafted work*: Rowena M.A. Packer, Eleanor Raffan, Anna Morros‐Nuevo and Nicole Regan. *Revised work*: Eleanor Raffan and Rowena M.A. Packer. *Founding acquisition*: Rowena M.A. Packer, Eleanor Raffan and Anna Morros‐Nuevo. *Writing*: Anna Morros‐Nuevo, Eleanor Raffan and Rowena M.A. Packer.

## CONFLICT OF INTEREST STATEMENT

The authors declare they have no conflicts of interest.

## ETHICS STATEMENT

This project was approved by the Social Science Research Ethical Review at the Royal Veterinary College, London, UK.

## Supporting information



Supporting Information

## Data Availability

The datasets generated and/or analysed during the current study are available in the Apollo, University of Cambridge repository (https://doi.org/10.17863/CAM.106981).

## References

[1] Kearsley‐Fleet L , O'Neill DG , Volk HA , Church DB , Brodbelt DC . Prevalence and risk factors for canine epilepsy of unknown origin in the UK. Vet Rec. 2013;172(13):338. 23300065 10.1136/vr.101133

[2] Berendt M , Farquhar RG , Mandigers PJJ , Pakozdy A , Bhatti SFM , De Risio L , et al. International veterinary epilepsy task force consensus report on epilepsy definition, classification and terminology in companion animals. BMC Vet Res. 2015;11:182. 10.1186/s12917-015-0461-2 PMC4552272 26316133

[3] Packer RMA , Shihab NK , Torres BBJ , Volk HA . Clinical risk factors associated with anti‐epileptic drug responsiveness in canine epilepsy. PLoS One. 2014;9(8):e106026. 25153799 10.1371/journal.pone.0106026 PMC4143335

[4] Nettifee JA , Munana KR , Griffith EH . Evaluation of the impacts of epilepsy in dogs on their caregivers. J Am Anim Hosp Assoc. 2017;53(3):143–149. 28291397 10.5326/JAAHA-MS-6537

[5] Packer RMA , De Risio L , Volk HA . Investigating the potential of the anti‐epileptic drug imepitoin as a treatment for co‐morbid anxiety in dogs with idiopathic epilepsy. BMC Vet Res. 2017;13(1):1–10. 28388948 10.1186/s12917-017-1000-0 PMC5383962

[6] Neßler J , Rundfeldt C , Löscher W , Kostic D , Keefe T , Tipold A . Clinical evaluation of a combination therapy of imepitoin with phenobarbital in dogs with refractory idiopathic epilepsy. BMC Vet Res. 2017;13(1):33. 28118828 10.1186/s12917-017-0957-z PMC5264332

[7] Chang Y , Mellor DJ , Anderson TJ . Idiopathic epilepsy in dogs: owners’ perspectives on management with phenobarbitone and/or potassium bromide. J Small Anim Pract. 2006;47(10):574–581. 17004949 10.1111/j.1748-5827.2006.00203.x

[8] Packer RMA , Shihab NK , Torres BBJ , Volk HA . Responses to successive anti‐epileptic drugs in canine idiopathic epilepsy. Vet Rec. 2015;176(8):203. 10.1136/vr.102934 25564473

[9] Kähn C , Meyerhoff N , Meller S , Nessler JN , Volk HA , Charalambous M . The postictal phase in canine idiopathic epilepsy: semiology, management, and impact on the quality of life from the owners’ perspective. Animals. 2024;14(1):103. 10.3390/ani14010103 PMC10778387 38200833

[10] Gilliam F . Optimizing health outcomes in active epilepsy. Neurology. 2002;58(8 Suppl. 5):S9–S20. 10.1212/wnl.58.8_suppl_5.s9 11971128

[11] Wessmann A , Volk HA , Packer RMA , Ortega M , Anderson TJ . Quality‐of‐life aspects in idiopathic epilepsy in dogs. Vet Rec. 2016;179(9):229. 27329504 10.1136/vr.103355

[12] Charalambous M , Brodbelt D , Volk HA . Treatment in canine epilepsy—a systematic review. BMC Vet Res. 2014;10:257. 25338624 10.1186/s12917-014-0257-9 PMC4209066

[13] Batcherlor DJ , German AJ . Chapter 7. Polyphagia. In: BSAVA manual of canine and feline gastroenterology. BSAVA; 2019. p. 46–48.

[14] Heymsfield SB , Avena NM , Baier L , Brantley P , Bray GA , Burnett LC , et al. Hyperphagia: current concepts and future directions proceedings of the 2nd international conference on hyperphagia. Obesity. 2014;22(Suppl. 1):S1–S17. 10.1002/oby.20646 PMC4159941 24574081

[15] Salt C , Morris PJ , Wilson D , Lund EM , German AJ . Association between life span and body condition in neutered client‐owned dogs. J Vet Intern Med. 2019;33(1):89–99. 30548336 10.1111/jvim.15367 PMC6335446

[16] Kealy RD , Lawler DF , Ballam JM , Mantz SL , Biery DN , Greeley EH , et al. Effects of diet restriction on life span and age‐related changes in dogs. J Am Vet Med Assoc. 2002;220(9):1315–1320. 11991408 10.2460/javma.2002.220.1315

[17] Lawler D , Evans R , Larson B , Spitznagel E , Ellersieck M , Kealy R . Influence of lifetime food restriction on causes, time, and predictors of death in dogs. J Vet Med Assoc. 2005;226(2):225–231. 10.2460/javma.2005.226.225 15706972

[18] Lawler DF , Larson BT , Ballam JM , Smith GK , Biery DN , Evans RH , et al. Diet restriction and ageing in the dog: major observations over two decades. Br J Nutr. 2008;99(4):793–805. 18062831 10.1017/S0007114507871686

[19] Podell M , Volk H , Berendt M , Loscher W , Munana K , Patterson E , et al. 2015 ACVIM small animal consensus statement on seizure management in dogs. J Vet Intern Med. 2016;30(2):477–490. 26899355 10.1111/jvim.13841 PMC4913615

[20] Morrison R , Penpraze V , Beber A , Reilly JJ , Yam PS . Associations between obesity and physical activity in dogs: a preliminary investigation. J Small Anim Pract. 2013;54(11):570–574. 24117778 10.1111/jsap.12142

[21] German AJ . The growing problem of obesity in dogs and cats. J Nutr. 2006;136(7 Suppl.):1940S‒1946S. 16772464 10.1093/jn/136.7.1940S

[22] Montoya‐Alonso JA , Bautista‐Castaño I , Peña C , Suárez L , Juste MC , Tvarijonaviciute A . Prevalence of canine obesity, obesity‐related metabolic dysfunction, and relationship with owner obesity in an obesogenic region of Spain. Front Vet Sci. 2017;4:59. 28487859 10.3389/fvets.2017.00059 PMC5403824

[23] Tvarijonaviciute A , Ceron JJ , Holden SL , Cuthbertson DJ , Biourge V , Morris PJ , et al. Obesity‐related metabolic dysfunction in dogs: a comparison with human metabolic syndrome. BMC Vet Res. 2012;8:147. 22929809 10.1186/1746-6148-8-147 PMC3514388

[24] Costa‐Santos K , Damasceno K , Portela RD , Santos FL , Araújo GC , Martins‐Filho EF , et al. Lipid and metabolic profiles in female dogs with mammary carcinoma receiving dietary fish oil supplementation. BMC Vet Res. 2019;15:401. 31703601 10.1186/s12917-019-2151-y PMC6839264

[25] Hill RC . Conference on ‘Multidisciplinary approaches to nutritional problems’. Symposium on ‘Nutrition and health’. Nutritional therapies to improve health: lessons from companion animals. Proc Nutr Soc. 2009;68(11):98–102. 19040782 10.1017/S0029665108008835

[26] Chandler M , Cunningham S , Lund EM , Khanna C , Naramore R , Patel A , et al. Obesity and associated comorbidities in people and companion animals: a one health perspective. J Comp Pathol. 2017;156(4):296–309. 28460795 10.1016/j.jcpa.2017.03.006

[27] German AJ , Hervera M , Hunter L , Holden SL , Morris PJ , Biourge V , et al. Improvement in insulin resistance and reduction in plasma inflammatory adipokines after weight loss in obese dogs. Domest Anim Endocrinol. 2009;37(4):214–226. 19674864 10.1016/j.domaniend.2009.07.001

[28] Jones GMC , Volk HA , Packer RMA . Research priorities for idiopathic epilepsy in dogs: viewpoints of owners, general practice veterinarians, and neurology specialists. J Vet Intern Med. 2021;35(3):1466–1479. 33960544 10.1111/jvim.16144 PMC8162594

[29] Ettinger AB , Carter JA , Rajagopalan K . Patient versus neurologist preferences: a discrete choice experiment for antiepileptic drug therapies. Epilepsy Behav. 2018;80:247–253. 29433949 10.1016/j.yebeh.2018.01.025

[30] Raffan E , Smith SP , O'Rahilly S , Wardle J . Development, factor structure and application of the dog obesity risk and appetite (DORA) questionnaire. PeerJ. 2015;1:e1278. 10.7717/peerj.1278 PMC4592153 26468435

[31] Wallis NJ , Sumanasekera NT , Raffan E . Obesity risk factors in British Labrador retrievers: effect of sex, neuter status, age, chocolate coat colour and food motivation. Vet Rec. 2024;194(6):e3410. 37747436 10.1002/vetr.3410

[32] German AJ , Holden SL , Moxham GL , Holmes KL , Hackett RM , Rawlings JM . A simple, reliable tool for owners to assess the body condition of their dog or cat. J Nutr. 2006;136(Suppl. 7):2031S‒2033S. 16772488 10.1093/jn/136.7.2031S

[33] Mawby DI , Bartges JW , d'Avignon A , Laflamme DP , Moyers TD , Cottrell T . Comparison of various methods for estimating body fat in dogs. J Am Anim Hosp Assoc. 2004;40(2):109–114. 15007045 10.5326/0400109

[34] Laflamme D . Development and validation of a body condition score system for dogs. Canine Practice. 1997;22(1):10–15.

[35] Raffan E , Dennis RJ , O'Donovan CJ , Becker JM , Scott RA , Smith SP , et al. A deletion in the canine POMC gene is associated with weight and appetite in obesity‐prone Labrador retriever dogs. Cell Metab. 2016;23(5):893–900. 27157046 10.1016/j.cmet.2016.04.012 PMC4873617

[36] Wallis N , Raffan E . The genetic basis of obesity and related metabolic diseases in humans and companion animals. Genes. 2020;11(11):1378. 33233816 10.3390/genes11111378 PMC7699880

[37] Bjørnvad CR , Gloor S , Johansen SS , Sandøe P , Lund TB . Neutering increases the risk of obesity in male dogs but not in bitches—a cross‐sectional study of dog‐ and owner‐related risk factors for obesity in Danish companion dogs. Prev Vet Med. 2019;170:104730. 31421500 10.1016/j.prevetmed.2019.104730

[38] Burns K . Golden Retriever study examining cancer, neuter status, obesity. American Veterinary Medical Association; 2016. [cited 21 Jul 2022]. Available from: https://www.avma.org/javma‐news/2016‐06‐01/golden‐retriever‐study‐examining‐cancer‐neuter‐status‐obesity

[39] Colliard L , Ancel J , Benet JJ , Paragon BM , Blanchard G . Risk factors for obesity in dogs in France. J Nutr. 2006;136(7 Suppl.):1951S‒1954S. 16772466 10.1093/jn/136.7.1951S

[40] Jeusette I , Detilleux J , Cuvelier C , Istasse L , Diez M . Ad libitum feeding following ovariectomy in female Beagle dogs: effect on maintenance energy requirement and on blood metabolites. J Anim Physiol Anim Nutr. 2004;88(3–4):117–121. 10.1111/j.1439-0396.2003.00467.x 15059235

[41] Lund EM , Armstrong PJ , Kirk CA , Klausner JS . Prevalence and risk factors for obesity in adult dogs from private US veterinary practices. J Appl Res Vet Med. 2006;4(2):177–186.

[42] Mao J , Xia Z , Chen J , Yu J . Prevalence and risk factors for canine obesity surveyed in veterinary practices in Beijing, China. Prev Vet Med. 2013;112 (3–4):438–442. 24042026 10.1016/j.prevetmed.2013.08.012

[43] McGreevy PD , Thompson PC , Pride C , Fawcett A , Grassi T , Jones B . Prevalence of obesity in dogs examined by Australian veterinary practices and the risk factors involved. Vet Rec. 2005;156(22):695‒702. 15923551 10.1136/vr.156.22.695

[44] Pegram C , Raffan E , White E , Ashworth AH , Brodbelt DC , Church DB , et al. Frequency, breed predisposition and demographic risk factors for overweight status in dogs in the UK. J Small Anim Pract. 2021;62(7):521‒530. 33754373 10.1111/jsap.13325

[45] Porsani MYH , Teixeira FA , Oliveira VV , Pedrinelli V , Dias RA , German AJ , et al. Prevalence of canine obesity in the city of São Paulo, Brazil. Sci Rep. 2020;10(1):14082. 32826948 10.1038/s41598-020-70937-8 PMC7442815

[46] Vendramini THA , Amaral AR , Pedrinelli V , Zafalon RVA , Rodrigues RBA , Brunetto MA . Neutering in dogs and cats: current scientific evidence and importance of adequate nutritional management. Nutr Res Rev. 2020;33(1):134–144. 31931899 10.1017/S0954422419000271

[47] Qualtrics Software [Internet]. Provo, UT, USA. Available from: https://www.qualtrics.com

[48] R studio team . RStudio: Integrated Development for R. RStudio, PBC. Boston, MA, USA: R studio [Internet]; 2020. Available from: http://www.rstudio.com/

[49] Faul F , Erdfelder E , Lang AG , Buchner A . G*Power 3: a flexible statistical power analysis program for the social, behavioral, and biomedical sciences. Behav Res Methods. 2007;39(2):175–191. 17695343 10.3758/bf03193146

[50] Faul F , Erdfelder E , Buchner A , Lang AG . Statistical power analyses using G*Power 3.1: tests for correlation and regression analyses. Behav Res Methods. 2009;41(4):1149–1160. 19897823 10.3758/BRM.41.4.1149

[51] Giner‐Sorolla R , Montoya AK , Reifman A , Carpenter T , Lewis NA Jr , Aberson CL , et al. Power to detect what? Considerations for planning and evaluating sample size. Personality Social Psychol Rev. 2024;28(3):276–301. 10.1177/10888683241228328 PMC11193916 38345247

[52] Cohen J . Chapter 2: the *t*‐test for means. Statistical power analysis for the behavioral sciences. 2nd ed. New York: Lawrence Erlbaum Associates; 1988.

[53] Charalambous M , Brodbelt D , Volk HA . The evidence behind the treatment of canine idiopathic epilepsy. Vet Evid. 2016;1(1). 10.18849/ve.v1i1.9

[54] Baird‐Heinz HE , Van Schoick AL , Pelsor FR , Ranivand L , Hungerford LL . A systematic review of the safety of potassium bromide in dogs. J Am Vet Med Assoc. 2012;240(6):705–715. 22380809 10.2460/javma.240.6.705

[55] Luño I , Palacio J , García‐Belenguer S , González‐Martínez Á , Rosado B . Emotional eating in companion dogs: owners’ perception and relation with feeding habits, eating behavior, and emotional state. J Vet Behav. 2018;25:17–23.

[56] Berk BA , Packer RMA , Law TH , Volk HA . Investigating owner use of dietary supplements in dogs with idiopathic epilepsy. Res Vet Sci. 2018;119:276–284. 30064067 10.1016/j.rvsc.2018.07.004

[57] Boothe DM , Dewey C , Carpenter DM . Comparison of phenobarbital with bromide as a first‐choice antiepileptic drug for treatment of epilepsy in dogs. J Am Vet Med Assoc. 2012;240(9):1073–1083. 22515627 10.2460/javma.240.9.1073

[58] Schwartz‐Porsche D , Löscher W , Frey HH . Therapeutic efficacy of phenobarbital and primidone in canine epilepsy: a comparison. J Vet Pharmacol Ther. 1985;8(2):113–119. 4020942 10.1111/j.1365-2885.1985.tb00934.x

[59] Podell M . Antiepileptic drug therapy. Clin Tech Small Anim Pract. 1998;13(3):185–192. 9775509 10.1016/S1096-2867(98)80040-6

[60] Dewey CW . Anticonvulsant therapy in dogs and cats. Vet Clin North Am Small Anim Pract. 2006;36(5):1107–1127. 16984829 10.1016/j.cvsm.2006.05.005

[61] Packer RMA , Volk HA , Fowkes RC . Physiological reactivity to spontaneously occurring seizure activity in dogs with epilepsy and their carers. Physiol Behav. 2017;177:27–33. 28412282 10.1016/j.physbeh.2017.04.008

[62] Rowland NE , Antelman SM . Stress‐induced hyperphagia and obesity in rats: a possible model for understanding human obesity. Science. 1976;191(4224):310–312. 1246617 10.1126/science.1246617

[63] Sandilands V , Tolkamp BJ , Savory CJ , Kyriazakis I . Behaviour and welfare of broiler breeders fed qualitatively restricted diets during rearing: are there viable alternatives to quantitative restriction? Appl Anim Behav Sci. 2006;96(1):53–67.

[64] Bazzigaluppi P , Ebrahim Amini A , Weisspapir I , Stefanovic B , Carlen PL . Hungry neurons: metabolic insights on seizure dynamics. Int J Mol Sci. 2017;18(11):2269. 29143800 10.3390/ijms18112269 PMC5713239

[65] Ladino LD , Téllez‐Zenteno JF . Chapter 7: epilepsy and obesity: a complex interaction. In: Mula M , editor. The comorbidities of epilepsy. Academic Press; 2019. p. 131–158.

[66] Barry M , Cameron S , Kent S , Barnes‐Heller H , Grady K . Daytime and nocturnal activity in treated dogs with idiopathic epilepsy compared to matched unaffected controls. J Vet Intern Med. 2021;35(4):1826–1833. 34223667 10.1111/jvim.16205 PMC8295678

[67] Pickup E , German AJ , Blackwell E , Evans M , Westgarth C . Variation in activity levels amongst dogs of different breeds: results of a large online survey of dog owners from the UK. J Nutr Sci. 2017;6:e10. 28620485 10.1017/jns.2017.7 PMC5465859

[68] Courcier EA , Mellor DJ , Thompson RM , Yam PS . A cross sectional study of the prevalence and risk factors for owner misperception of canine body shape in first opinion practice in Glasgow. Prev Vet Med. 2011;102(1):66‒74. 21820746 10.1016/j.prevetmed.2011.06.010

[69] Eastland‐Jones RC , German AJ , Holden SL , Biourge V , Pickavance LC . Owner misperception of canine body condition persists despite use of a body condition score chart. J Nutr Sci. 2014;3:e45. 26101613 10.1017/jns.2014.25 PMC4473163

[70] Naughton V , Grzelak T , Mulhern M , Moffett R , Naughton P . Caring practices of pet cat and dog owners in Northern Ireland vs potential implications for animals’ health and welfare. Anim Welf. 2021;30(2):131–144.

[71] Rolph NC , Noble PJM , German AJ . How often do primary care veterinarians record the overweight status of dogs? J Nutritional Sci. 2014;3(e58):1–5. 10.1017/jns.2014.42 PMC4473162 26101626

[72] White GA , Hobson‐West P , Cobb K , Craigon J , Hammond R , Millar KM . Canine obesity: is there a difference between veterinarian and owner perception? J Small Anim Pract. 2011;52(12):622–626. 22017760 10.1111/j.1748-5827.2011.01138.x

[73] Segami CL , Dávila FR , Lira MB , Segami CL , Dávila FR , Lira MB . Factores asociados a la obesidad en perros adultos de Lima, Perú. Rev Investig Vet. 2021;32(3):e20399..

[74] Slater MR , Robinson LE , Zoran DL , Wallace KA , Scarlett JM . Diet and exercise patterns in pet dogs. J Am Vet Med Assoc. 1995;207(2):186–190. 7601712

[75] Fukuoka Y , Haskell W , Vittinghoff E . New insights into discrepancies between self‐reported and accelerometer‐measured moderate to vigorous physical activity among women—the mPED trial. BMC Public Health. 2016;16(1):1–11. 27514368 10.1186/s12889-016-3348-7 PMC4982411

