# Supplementary material for: Caregiver‐reported increased food motivation and adiposity in dogs receiving antiseizure drugs
Source: Vet Rec. 2024 Dec 10;195(12):e4907. doi: 10.1002/vetr.4907 (PMC11639269; doi:10.1002/vetr.4907)
Supplement: Supplementary file 1 — Supporting Information [file VETR-195-e4907-s001.docx]

# SUPPLEMENTARY MATERIAL

# Manuscript ‘Increased food motivation and adiposity based on caregiver survey in dogs receiving anti-seizure drugs

## 1.- SELECTION OF CONTROL GROUP

Subjects for the control group were obtained from an existing database containing over 19,000 dogs. Only dogs 1-20 years old with no chronic or recurrent health conditions with potential (known or suspected effect) to modify eating behaviour/appetite, level of activity/general demeanour or weight status or who were not taking medications that could impact weight, hunger or activity levels were included.

Where medical history was available, this was used to determine if the criteria applied. Where medical history was not available, multiple-choice answers about organ system diseases and free text. The options available were:

- Endocrine (e.g. hypothyroidism, adrenal disease)
- Musculoskeletal: Exclude animals on joint supplements who have not specified that the animal has an orthopaedic condition (e.g. hip dysplasia, spondylosis).
- Oral (e.g. oral mass, severe dental diseases).
- Behaviour (e.g. cognitive dysfunction, generalised anxiety or phobia)
- Gastrointestinal: Pancreatic diseases is included here. (e.g. chronic pancreatitis, megaoesophagus, IBD).
- Neoplasia: Excluded diagnosed benign and non-invasive masses (e.g. lymphoma, osteosarcoma).
- Cardiorespiratory (e.g. MMVD, CHF, tracheal collapse, laryngeal paralysis).
- Neurological: Excludes cognitive dysfunction (e.g. Idiopathic epilepsy, Wobblers syndrome, Syringomyelia)
- Medication: Animals on long-term or recurrent medications with known or cited side effects of appetite, level of activity or weight (e.g. steroid anti-inflammatories).
- Dermatology (e.g. alimentary allergies)
- Renal (e.g. CKD).
- Ophthalmic (e.g. glaucoma, sudden or congenital blindness).

Respondents’ answers and free text were assessed through the following steps:

1. Diagnostic terms identified (e.g. hypothyroidism, elbow dysplasia, atopic dermatitis).
2. Symptomatology described (e.g. seizures, excessive drinking, heart murmur).
3. Lay terms compatible with diseases or symptoms of interest (e.g. itching every summer, fits).

New categories were created to accommodate information obtained from the free text answers:

- Other significant: any other ‘condition’ that is expected to affect appetite, weight or level of energy and stamina in the long term and that does not fall into any of the previous categories. Includes animals on "special diets" (raw diet, veterinary prescription diets, meet-free diet, grain and gluten free diet, etc.). Hepatopathies and splenic disease are included here. Animals reported to have been rehomed less than one year prior to responding and that had severe medical conditions that could have affected their appetite, weight or level of activity (specially animals who have been rescued): emaciation, malnourishment, etc were also excluded.
- Other non-significant: Includes any condition or medication or supplements mentioned that is not expected to affect appetite, weight or level of activity in the long term (e.g. regular flea and worm treatment).

## 2.- QUESTIONNAIRES

## 2.1.- DORA questionnaire

The DORA questionnaire (Raffan et al., 2015) contains 34 statements which are used to obtain 4 owner management subfactors (‘owner perception’, ‘owner intervention’, ‘restriction of human food’ and ‘exercise’) and 3 dog behaviour subfactors (‘Interest in food’, ‘lack of fussiness’ and ‘Responsiveness and satiety’). Each statement is a 4- or 5-point Likert scale and the results are converted into numeric 1-4 (‘Not at all true’=1, ‘Somewhat true’=2, ‘Mainly true’=3, ‘Definitely true’=4) or 1-5 respectively (‘Never’=1, ‘Rarely’=2, ‘Sometimes’=3, ‘Often’=4, ‘Always’=5). Before factor calculation, some statements’ scores are reversed. For example, for a statement like ‘my dog would eat anything’, the higher the score, the higher the agreement, while the statement ‘my dog takes his/her time to eat a meal’, the higher the score the least eagerly the dog eats, so this score is reversed for ease of interpretation. In the original paper, each factor is scores from 0 to 100, we used a 0-1 scoring system because we felt it was easier to interpret. For our study, we were interested in all three dog subfactors and all ‘owner management subfactor’ except for ‘owner perception’. The remaining of ‘owner management subfactors’ were used to obtain ‘owner control’ score by calculating the mean of the statements included in ‘owner intervention’, ‘restriction of human food’ and ‘exercise’ subfactors. The questionnaire also provides subfactors ‘Current disease score’ and ‘Signs of Gastrointestinal Disease Scores’, that we did not use for this study.

| **Owner Management** |
| --- |
| Owner Perception Score |
| 1. My dog is very fit (reversed) 2. I think my dog could do with losing some weight. 3. I am happy with my dog’s weight (reversed). |
| Owner Control Score |
| *Owner Intervention Score* |
| 1. I alter the food my dog gets in order to control his/her weight. 2. I am careful about my dog’s weight. 3. I weight or measure how much food I give my dog. 4. I am careful to regulate the exercise my dog gets in order to keep him/her slim. |
| *Restriction of Human Food Score* |
| 1. My dog gets bits of human food when we are eating (reversed). 2. My dog often gets human food (reversed). 3. My dog gets no food at human mealtimes. 4. My dog gets human leftovers in his/her food bowl (reversed). |
| *Exercise Score* |
| 1. My dog spends most of his/her walks off the lead. 2. My dog runs around a lot. 3. My dog walks mostly on the lead (reversed). 4. My dog walks involve a lot of energetic play or chasing. 5. My dog gets a lot of exercise. |
| **Dog Food Motivation Score** |
| *Responsiveness and Satiety Score* |
| 1. My dog will turn food down if he/she is not hungry (reversed). 2. My dog gets excited when there is food around. 3. My dog finishes a meal straight away. 4. After a meal, my dog is still interested in eating. 5. My dog takes his/her time to eat a meal (reversed). 6. My dog seems to be hungry all the time. 7. My dog is very greedy. |
| *Lack of Fussiness Score* |
| 1. My dog inspects unfamiliar food before deciding whether to eat them (reversed). 2. My dog is choosy about which titbits he/she eats (reversed). 3. My dog would eat anything. |
| *Interest in Food Score* |
| 1. My dog hangs around for titbits even if there is not much chance of getting them. 2. My dog hangs around when I am preparing or eating human food. 3. My dog eats titbits straight away. |
| **Signs of Current Disease** |
| 1. My dog gets an upset tummy on some foods. 2. My dog has a sensitive stomach. 3. My dog often gets tummy upsets. |
| **Current disease** |
| 1. My dog regularly sees the vet for health problems. 2. I restrict my dog’s exercise because of veterinary advice. |

## 2.2.- ASD impact on behaviour

Current ASD treatment information was collected, including ASDs currently used, dose for each ASD, length of treatment and perceived treatment efficiency. Owners were asked if they believed that their dogs current treatment had: reduced their energy to play (lethargy), reduced their control/coordination (Ataxia), increased their appetite (hunger), or increased their food intake (polyphagia). These possible answers were ‘Not at all true’, ‘Somewhat true’, ‘Mainly true’ and ‘Definitely true’. Owners who previously reported that their dog is treated with multiple ASDs, were then asked which ASD they believed had the biggest impact on their dog’s appetite.

## 3.- BCS VISUAL SCALE

#
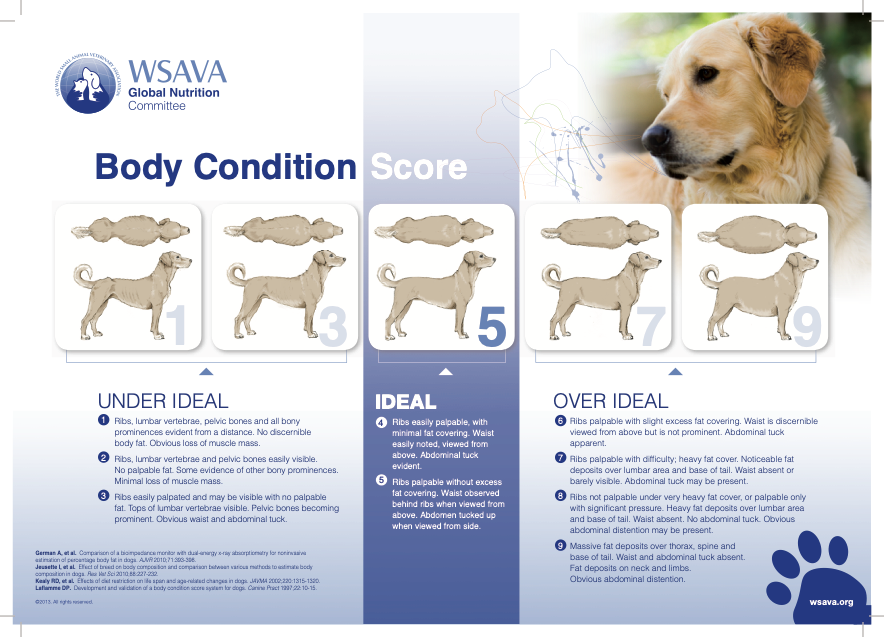


Available at <https://wsava.org/wp-content/uploads/2020/01/Body-Condition-Score-Dog.pdf>

## 4.- LINEAR REGRESSION ANALYSIS

To assess the combined effect of sex, age, neuter status and ASD on Food Motivation Score within the epileptic group, minimal modelling was performed using Akaike’s Information Criterion (AIC). Categorical variables were converted to binomial: sex (0=Female, 1=Male), neuter status (0=Entire, 1=Neutered) and ASD (0=No, 1=Yes). Age remained as a continuous variable.

The first model fitted were as follows:

*Model 1: FMS ~ Sex*NeuterStatus + Sex*Age + NeuterStatus*Age*

*Minimal model (1a):FMS ~ Sex*NeuterStatus + NeuterStatus*Age*

Following obtention of the minimal model, ASD was added to generate a second model:

*Model 2: FMS~ Sex*NeuterStatus + NeuterStatus*Age + ASD*

*Minimal model (2a): FMS ~ Sex*NeuterStatus + ASD*

The resulting minimal models were compared using effect size (β), significance level (p) and variability explained (R^2^) (Table 1).

**Table 1. Significant results obtained from the minimal models are shown.** For each variable, effect size β and significance (p-value) are shown. Level of significance is set at p<0.05 (‘ns’ indicates ‘not significant’). Where binomial variables have been used, the direction of the β-value (negative or positive) indicates the direction of the effect. For example, ASD has a β-value of 0.32, which indicates that receiving ASD (1=Yes; 0=No) has the potential to increment FMS by 0.32%.

|  | **Model 1a** | **Model 2a** |
| --- | --- | --- |
| **Sex** | -0.05 (0.001) | ns |
| **Neuter status** | ns | -0.17 (0.042) |
| **Age** | - | - |
| **Sex : Age** | - | - |
| **Sex : Neuter Status** | 0.05 (<0.001) | 0.22 (0.012) |
| **Age : Neuter Status** | - | - |
| **ASD** | - | 0.32 (<0.001) |
| **Variability explained** | 0.018 | 0.089 |

## 5.- BREED DISTRIBUTION

**Table 2. Border collies were overrepresented in the epileptic group besides breed-matching process.** The number of dogs and upward-rounded percentage of each purebred, crossbreed and ‘other breeds’ in each group are shown. The numbers obtained for the epileptic group are those obtained through recruitment of participants. The control group was matched as close as possible by percentage of each breed from an existing database of over 19,000 dogs.

|  | **Control** | | **Epileptics** | |
| --- | --- | --- | --- | --- |
|  | Count | Percentage | Count | percentage |
| **Cross** | 1782 | 25.15 | 55 | 24.77 |
| **Border Collie** | 458 | 6.46 | 37 | 16.67 |
| **Other Purebred** | 517 | 7.30 | 24 | 10.81 |
| **English Springer spaniel** | 176 | 2.48 | 7 | 3.15 |
| **Golden retriever** | 423 | 5.97 | 6 | 2.70 |
| **Labrador retriever** | 161 | 2.27 | 6 | 2.70 |
| **English Cocker spaniel** | 374 | 5.28 | 5 | 2.25 |
| **Australian shepherd** | 192 | 2.71 | 4 | 1.80 |
| **French bulldog** | 272 | 3.84 | 4 | 1.80 |
| **German shepherd** | 292 | 4.12 | 4 | 1.80 |
| **Hungarian Vizsla** | 0 | 0.00 | 4 | 1.80 |
| **Siberian Husky** | 40 | 0.56 | 4 | 1.80 |
| **Staffordshire Bull terrier** | 219 | 3.09 | 4 | 1.80 |
| **Beagle** | 113 | 1.59 | 3 | 1.35 |
| **Boston terrier** | 70 | 0.99 | 3 | 1.35 |
| **Patterdale terrier** | 21 | 0.30 | 3 | 1.35 |
| **American Cocker spaniel** | 24 | 0.34 | 2 | 0.90 |
| **Bichon Frise** | 56 | 0.79 | 2 | 0.90 |
| **Bull terrier** | 34 | 0.48 | 2 | 0.90 |
| **Chihuahua** | 44 | 0.62 | 2 | 0.90 |
| **Cockapoo** | 11 | 0.16 | 2 | 0.90 |
| **Dalmatian** | 138 | 1.95 | 2 | 0.90 |
| **English bulldog** | 133 | 1.88 | 2 | 0.90 |
| **German Shorthaired pointer** | 52 | 0.73 | 2 | 0.90 |
| **Greyhound** | 76 | 1.07 | 2 | 0.90 |
| **Irish setter** | 13 | 0.18 | 2 | 0.90 |
| **Jack Russell terrier** | 226 | 3.19 | 2 | 0.90 |
| **Keeshond** | 8 | 0.11 | 2 | 0.90 |
| **Miniature Poodle** | 67 | 0.95 | 2 | 0.90 |
| **Nova Scotia Duck Tolling retriever** | 16 | 0.23 | 2 | 0.90 |
| **Rottweiler** | 49 | 0.69 | 2 | 0.90 |
| **Standard Poodle** | 141 | 1.99 | 2 | 0.90 |
| **Alaskan Malamute** | 14 | 0.20 | 1 | 0.45 |
| **American bulldog** | 48 | 0.68 | 1 | 0.45 |
| **Basset hound** | 76 | 1.07 | 1 | 0.45 |
| **Boxer** | 50 | 0.71 | 1 | 0.45 |
| **Cavalier King Charles spaniel** | 74 | 1.04 | 1 | 0.45 |
| **Dachshund** | 64 | 0.90 | 1 | 0.45 |
| **German Wirehaired pointer** | 62 | 0.87 | 1 | 0.45 |
| **Labradoodle** | 15 | 0.21 | 1 | 0.45 |
| **Miniature pinscher** | 78 | 1.10 | 1 | 0.45 |
| **Miniature schnauzer** | 72 | 1.02 | 1 | 0.45 |
| **Pug** | 71 | 1.00 | 1 | 0.45 |
| **Rhodesian Ridgeback** | 21 | 0.30 | 1 | 0.45 |
| **Shih Tzu** | 63 | 0.89 | 1 | 0.45 |
| **Standard schnauzer** | 52 | 0.73 | 1 | 0.45 |
| **West Highland White Terrier** | 0 | 0.00 | 1 | 0.45 |
| **Whippet** | 70 | 0.99 | 1 | 0.45 |
| **Yorkshire terrier** | 58 | 0.82 | 1 | 0.45 |
|  | 7086 | 100 | 222 | 100 |

## 6.- DESCRIPTIVE STATISTICS AND T-TESTs

**Table 3. Mean and standard deviation (mean +/- SD) are shown for all DORA factors and subfactors.** Data from the epileptic group is shown in accordance to ASD status (drug naïve, receiving monotherapy and receiving polytherapy).

|  | | **Epileptic group** | | | |
| --- | --- | --- | --- | --- | --- |
|  | **Control group** | **Drug Naïve** | **All dogs receiving ASD** | **Dogs receiving 1 ASD** | **Dogs receiving >1 ASD** |
| **Number of dogs** | 7,086 | 8 | 214 | 80 | 134 |
| **Age (years)** | 5.50 +/- 3.58 | 7.5 +/- 2.98 | 5.6 +/- 4.82 | 5.7 +/- 7.19 | 5.49 +/- 2.54 |
| **Body Condition Score** | 4.82 +/- 0.99 | 4.5 +/- 0.53 | 5.28 +/- 1.36 | 5.12 +/- 1.06 | 5.37 +/- 1.51 |
| **Food Motivation Score** | 0.58 +/- 0.25 | 0.42 +/- 0.31 | 0.72 +/- 0.23 | 0.69 +/- 0.25 | 0.73 +/- 0.22 |
| **Responsiveness and Satiety** | 0.58 +/- 0.28 | 0.47 +/- 0.37 | 0.72+/- 0.24 | 0.71 +/- 0.26 | 0.74 +/- 0.23 |
| **Lack of Fussiness** | 0.52 +/- 0.31 | 0.29 +/- 0.36 | 0.68 +/- 0.29 | 0.64 +/- 0.31 | 0.69 +/- 0.28 |
| **Interest in Food** | 0.66 +/- 0.26 | 0.44 +/- 0.24 | 0.74 +/- 0.26 | 0.73 +/- 0.26 | 0.75 +/- 0.26 |
| **Owner Control** | 0.62 +/- 0.16 | 0.70 +/- 0.19 | 0.64 +/- 0.16 | 0.63 +/- 0.17 | 0.64 +/- 0.16 |
| **Owner Intervention** | 0.58 +/- 0.27 | 0.65 +/- 0.31 | 0.64 +/- 0.25 | 0.62 +/- 0.27 | 0.66 +/- 0.24 |
| **Restriction of Human Food** | 0.64 +/- 0.25 | 0.72 +/- 0.26 | 0.71 +/- 0.24 | 0.68 +/- 0.25 | 0.72 +/- 0.24 |
| **Exercise** | 0.65 +/- 0.24 | 0.74 +/- 0.24 | 0.56 +/- 0.27 | 0.59 +/- 0.28 | 0.54 +/- 0.27 |

**Table 4.** Pair-wise t-test with ‘holm’ correction comparing age (years), Body Condition Score and DORA scores between control group and epileptic dogs with and without medication (ASD). Cells contain p-value rounded upwards.

|  | **Control vs**  **Drug-naive Epileptics** | **Control vs**  **Medicated Epileptics** | **Epileptics: Drug-naive vs Medicated** | **Epileptics: Monotherapy vs Polytherapy** |
| --- | --- | --- | --- | --- |
| **Age** | 0.36 | 0.78 | 0.36 | 1.00 |
| **Body Condition Score** | 0.371 | 1.9e-10 | 0.064 | 0.247 |
| **Food Motivation** | 0.0673 | 4.8e-14 | 0.0021 | 0.3513 |
| **Responsiveness and Satiety** | 0.277 | 7.3e-14 | 0.022 | 0.55498 |
| **Lack of Fussiness** | 0.036 | 6.5e-13 | 0.001 | 0.2354 |
| **Interest in Food** | 0.0119 | 3.9e-05 | 0.0019 | 0.48616 |
| **Owner Control** | 0.51 | 0.51 | 0.51 | 1.00 |
| **Owner Intervention** | 0.9502 | 0.0024 | 0.9561 | 1.00 |
| **Restriction Human Food** | 0.70369 | 0.00011 | 0.90461 | 0.98265 |
| **Exercise** | 0.309 | 3e-07 | 0.085 | 0.33 |

**Table 5. Most respondents always use treats to administer ASD but do not compensate by reducing the dog’s main ration.** Table shows the number of participants (upward-rounded percentage in brackets) that reported to ‘Never’, ‘Rarely’, ‘Sometimes’ ‘Often’ or ‘Always’ give extra treats to their dogs because they have epilepsy, because they are going or have had a seizure or to administer ASD and that alter meals to compensate for treats.

|  | **Never** | **Rarely** | **Sometimes** | **Often** | **always** |
| --- | --- | --- | --- | --- | --- |
| **Do you give your dog extra treats or food because she/he have epilepsy?** | 156 (70.1%) | 25 (11.4%) | 24  (10.8%) | 11  (5%) | 6 (2.7%) |
| **Do you give your dog extra treats because she/he are going to have a seizure?** | 159 (71.6%) | 30 (13.5%) | 19  (8.6%) | 6 (2.7%) | 8 (3.6%) |
| **Do you give your dog treats because she/he had a seizure?** | 89 (40%) | 36 (16.2%) | 35  (15.8%) | 21 (9.5%) | 41 (18.5%) |
| **Do you use treats to administer the treatment?** | 43 (19.3%) | 21 (9.5%) | 18  (8.1%) | 22 (9.9%) | 118 (53.2%) |
| **Do you alter meals to compensate for treats?** | 75 (33.8%) | 34 (15.3%) | 45  (20.3%) | 36 (16.2%) | 32 (14.4%) |

**Table 6. Most respondents considered increased hunger as a side effect of ASD but not an increase in food intake.** Table show the number of participants (upward-rounded percentage in brackets) that considered to be ‘Not at all true’, ‘Somewhat true’, ‘Mainly true’ or ‘Definitely true’ to that ASD resulted in a decrease of their dog’s coordination, decreased level of energy to play, increased hunger or increased food intake.

|  | **Definitely true** | **Mainly true** | **Somewhat true** | **Not at all true** |
| --- | --- | --- | --- | --- |
| **Decreased coordination** | 43 (19.6%) | 20 (9.1%) | 71 (32.5%) | 85 (38.8%) |
| **Decreased energy** | 44 (20.1%) | 24 (10.9%) | 63 (28.8%) | 88 (40.2%) |
| **Increased hunger** | 109 (49.5%) | 37 (16.8%) | 42 (19%) | 32 (14.5%) |
| **Increased food intake** | 57 (26.1%) | 32 (14.7%) | 59 (27%) | 70 (32.1%) |
